# Experimental Effects of Acute Exercise on Iconic Memory, Short-Term Episodic, and Long-Term Episodic Memory

**DOI:** 10.3390/jcm7060146

**Published:** 2018-06-11

**Authors:** Danielle Yanes, Paul D. Loprinzi

**Affiliations:** Exercise Psychology Laboratory, Physical Activity Epidemiology Laboratory, Department of Health, Exercise Science and Recreation Management, The University of Mississippi, Oxford, MS 38677, USA; dmyanes@go.olemiss.edu

**Keywords:** memory consolidation, memory encoding, physical activity, visual memory

## Abstract

The present experiment evaluated the effects of acute exercise on iconic memory and short- and long-term episodic memory. A two-arm, parallel-group randomized experiment was employed (*n* = 20 per group; M_age_ = 21 year). The experimental group engaged in an acute bout of moderate-intensity treadmill exercise for 15 min, while the control group engaged in a seated, time-matched computer task. Afterwards, the participants engaged in a paragraph-level episodic memory task (20 min delay and 24 h delay recall) as well as an iconic memory task, which involved 10 trials (at various speeds from 100 ms to 800 ms) of recalling letters from a 3 × 3 array matrix. For iconic memory, there was a significant main effect for time (F = 42.9, *p* < 0.001, η^2^*_p_* = 0.53) and a trend towards a group × time interaction (F = 2.90, *p* = 0.09, η^2^*_p_* = 0.07), but no main effect for group (F = 0.82, *p* = 0.37, η^2^*_p_* = 0.02). The experimental group had higher episodic memory scores at both the baseline (19.22 vs. 17.20) and follow-up (18.15 vs. 15.77), but these results were not statistically significant. These findings provide some suggestive evidence hinting towards an iconic memory and episodic benefit from acute exercise engagement.

## 1. Introduction

Human memory is complex and can be categorized into multiple memory systems including sensory memory (very short term memory recall from various senses such as visual or auditory), working memory (recall of information while concurrently processing other information), prospective memory (remembering to perform a task in the future) and short- and long-term memory [1,2]. With the latter memory systems, declarative memory involves the recall of past events/episodes (episodic memory) or facts (semantic memory), whereas implicit or procedural memory involves memories that are not consciously encoded.

Of interest to the present experiment is sensory memory [3,4,5,6,7,8,9] and short- and long-term episodic memory. Visual memory systems can store information for a few hundred milliseconds, which can then be transferred to the limited capacity of the working memory system. Although controversial [10,11,12], this visual sensory (iconic) memory system is thought to heavily rely on cognitive attention [13,14].

Among the young adult population, most of the research examining the effects of acute exercise on episodic memory function have focused on short-term memory, with little research focusing on longer-term memory [15]. Thus, additional research evaluating the effects of acute exercise on long-term episodic memory function is needed. Research has demonstrated that acute exercise can enhance memory function [16,17,18,19,20,21], with mechanisms discussed elsewhere [1,22,23,24,25]. Briefly, postulated mechanisms include, for example, (1) exercise enhancing neuronal excitability; (2) exercise enhancing attentional resource allocation to facilitate memory encoding; (3) exercise upregulating AMPA receptor levels, opening NMDA channels, and increasing EPSP (excitatory post-synaptic potentials) in the hippocampus; (4) exercise priming neurons to be encoded in the memory trace by increasing CREB transcription; (5) BDNF (brain-derived neurotropic factor) expression from exercise; and (6) exercise enhancing dendritic spine growth [1].

In the context of iconic memory, exercise may, in theory, influence memory via exercise-induced changes in attention [16]. Some of the same brain structures (e.g., prefrontal cortex, parietal cortex) subserving attentional focus have been shown to be activated with acute exercise [26,27,28,29,30,31,32,33,34,35,36,37,38,39]. No study, to our knowledge, has examined the effects of acute exercise on iconic memory. Thus, the purpose of this experiment, written as a brief report, was to examine the effects of acute exercise on iconic memory and both short- and long-term episodic memory. We hypothesized that acute exercise would favorably influence both iconic memory as well as short- and long-term episodic memory. We speculated that acute exercise may enhance iconic memory via psychological attentional-based mechanisms, and also hypothesized that, as discussed above, episodic memorial effects from exercise may occur from alterations in key neurotransmitters involved in the consolidation of episodic memories.

## 2. Methods

### 2.1. Study Design

An experimental design was employed. Specifically, a two-arm, parallel-group randomized controlled intervention was utilized. Participants were randomized into either an experimental group or a control group. The experimental group was asked to walk briskly for 15 min on a treadmill while the control group engaged in a time-matched, seated task that involved playing an online game. Afterwards, the participants commenced the two memory tasks (episodic and iconic). Additional details are provided below. See Supplementary Materials for a schematic of the study design and procedures.

### 2.2. Participants

Each group included 20 participants (*n* = 40). This was based on our previous experimental work on this topic, demonstrating adequate statistical power for other related projects [16,17,18,40]. Recruitment occurred via a convenience-based, non-probability sampling approach. Participants were recruited from the authors’ university including both undergraduate and graduate students, ranging in age from 18–35 years. Participants were excluded based on the following criteria:

- Self-reported being a daily smoker [41,42]
- Self-reported being pregnant [43]
- Had exercised within 5 h of laboratory testing [15]
- Consumed any caffeine within 3 h of laboratory testing [44]
- Self-reported a concussion or head trauma within the past 30 days [45]
- Self-reported taking marijuana or other illegal drugs within the past 30 days [46]
- Were considered a daily alcohol user (>30 drinks/month for women; >60 drinks/month for men) [47]

### 2.3. Exercise Protocol

The treadmill bout of exercise involved walking on a treadmill for 15 min at a self-selected “brisk walking pace”. They were instructed to walk at a pace (minimum of 3.0 mph) as if they were late for catching a bus. This exact exercise protocol has previously been shown to enhance episodic memory performance [40]. The bout of exercise occurred prior to the initial memory assessment. Immediately after the bout of exercise, participants rested in a seated position for 5 min. After this resting period, they started the memory assessments, as described below.

### 2.4. Control Protocol

Those randomized to the control group completed a medium-level, online administered, Sudoku puzzle. Participants completed this puzzle for 20 min prior to completing the memory task (described below). The website for this puzzle is located at https://www.websudoku.com.

### 2.5. Memory Assessments and Procedures

Supplementary Materials present a schematic of the experimental protocol. After the exercise or control conditions, participants read a six-paragraph (374 words) passage used in other episodic memory research [48]. Prior to reading this passage, participants were told to read the passage carefully as, later in the experiment, they would be asked to try and recall as much information from this story as possible. Supplementary Materials also present the details of the scoring rubric that was used for this paragraph-level, episodic memory assessment. The maximum score was 42, with one point given for each aspect of the story recalled. A higher score indicated greater episodic memory function.

After reading the passage once, participants watched a 5-min video (The Office Bloopers—Season 3) then commenced the iconic memory assessment. During the assessment, participants sat 48 cm from the display monitor, where their head was stabilized by a chinrest. At this viewing distance, a 3 × 3 letter array appeared. Participants completed three practice sessions. Following these practice sessions, and as noted in Supplementary Materials, each whole-report iconic assessment included a “+” symbol (1000 ms) followed by the display of the 3 × 3 letter array. This array was displayed for 100 ms, 200 ms, 300 ms, 500 ms, or 800 ms (random order). Two trials for each speed occurred. Thus, 10 iconic trials occurred (two at 100 ms, two at 200 ms, two at 300 ms, two at 500 ms, and two at 800 ms). Immediately after each trial, participants were asked to write down as many letters as they could recall, and then write them down in the order in which they appeared in the 3 × 3 array. The main outcome for this task (for each interval speed) was the number of correct responses. A response was classified as correct if the participant reported the correct letter in the correct position in the array. Additionally, errors were also calculated. A mislocation error was defined as a reported letter that had been present in the array, but not in the position that was indicated by the participant. An intrusion error was defined as a reported letter that was not present in the array.

After the 10 iconic memory trials, participants watched another 5-min video (The Office Bloopers—Season 4). Following this video, and for the short-term episodic memory assessment (20-min delay), they were asked to recall as much information as possible from the six-paragraph passage (Supplementary Materials). Participants wrote down as much information from this passage as they could remember. This concluded the first laboratory assessment. For the long-term episodic memory assessment, participants returned to the laboratory approximately 24 h (range, 22–26 h) after their first laboratory visit. Upon arrival, they sat for 5-min and then re-completed the episodic memory assessment by writing down as much information from the story that they could remember.

### 2.6. Additional Assessments

Various other assessments were completed (at the beginning of the visit) to ensure that the two groups were similar in these parameters. As a measure of habitual physical activity behavior, and reported as time spent per week in moderate–to–vigorous physical activity (MVPA), participants completed a survey (Physical Activity Vital Signs Questionnaire) [49]. Waist circumference was measured to provide anthropometric characteristics of the sample. Finally, before, during, and after the exercise and control conditions, heart rate (chest-strapped Polar monitor, F1 model) was assessed.

### 2.7. Statistical Analysis

All statistical analyses were computed in SPSS (v. 24). For the iconic memory assessment, a 2 (group) × 10 (trials) repeated measures ANOVA was computed. For the episodic memory assessment, a 2 (group) × 2 (time) repeated measures ANOVA was computed. Statistical significance was set at an alpha of 0.05. Partial eta-squared (η^2^*_p_*) was calculated as a measure of effect size.

## 3. Results

Table 1 displays the characteristics of the sample. Across both groups, participants were similar regarding age, gender, race, waist circumference, and engagement in habitual exercise. Resting heart rates were similar across the two groups, and as expected, heart rate increased substantively during exercise (from 74 to 127 bpm).

**Figure 1:**
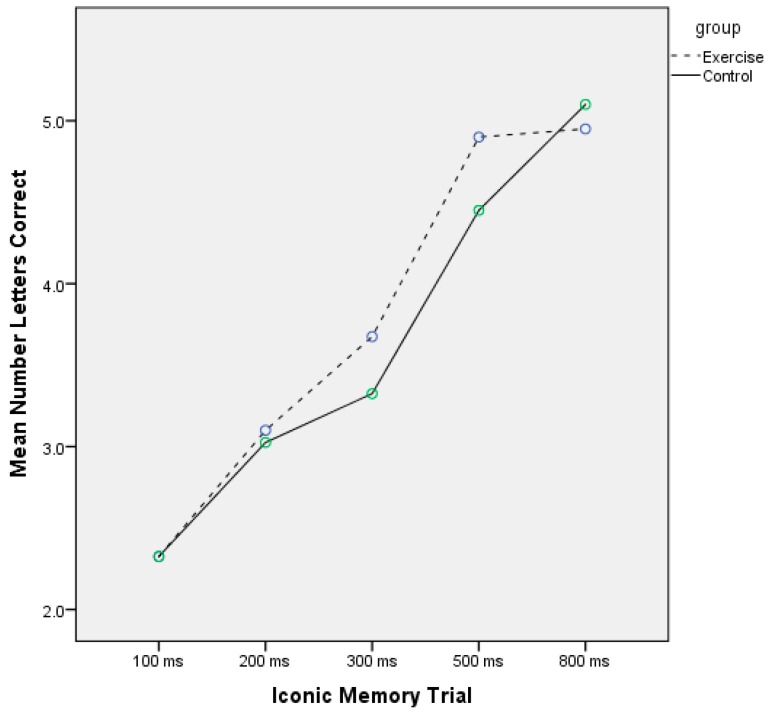
Iconic memory performance (absolute correct) across the experimental and control groups. Scores are the averages for each of the two trials for each temporal period. Results are presented from the shortest (100 ms) to longest (800 ms) iconic trials (in the experiment, trials were counterbalanced).

**Figure 2:**
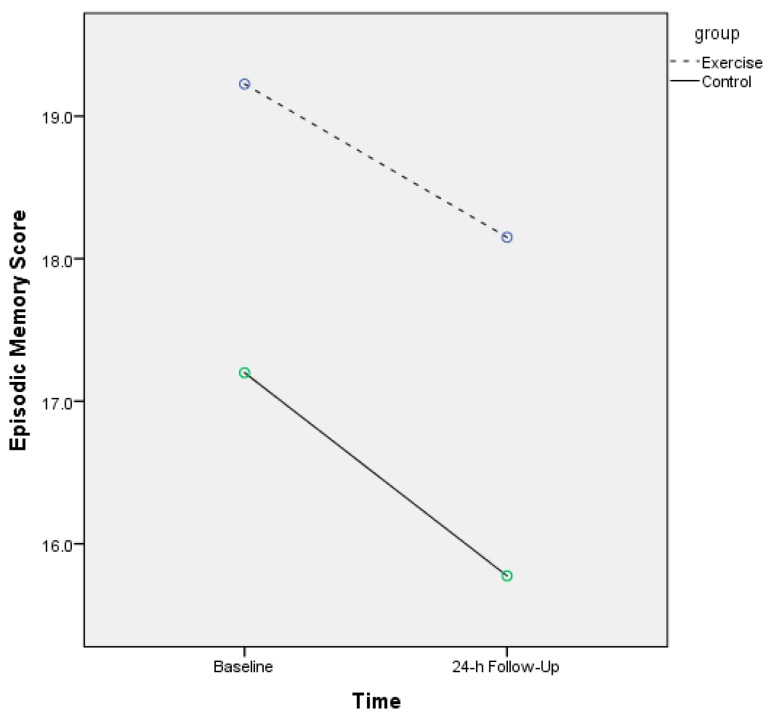
Episodic memory function scores across the experimental and control groups.

Table 2 displays the iconic and episodic memory scores for both groups. For iconic memory, and for absolute correct, there was a significant main effect for time (F = 42.9, *p* < 0.001, η^2^*_p_* = 0.53) and a trend toward a linear group–time interaction (F = 2.90, *p* = 0.09, η^2^*_p_* = 0.07), but no main effect for group (F = 0.82, *p* = 0.37, η^2^*_p_* = 0.02). Figure 1 displays the mean absolute correct scores for both groups. For most iconic trials, the exercise group (vs. control group) had a higher absolute correct score. For the mislocation iconic error assessment, there was no significant main effect for time (F = 1.56, *p* = 0.12, η^2^*_p_* = 0.04) or a significant group–time interaction (F = 0.97, *p* = 0.46, η^2^*_p_* = 0.02). Similarly, for intrusion errors, there was no significant main effect for time (F = 1.20, *p* = 0.29, η^2^*_p_* = 0.03) or a significant group–time interaction (F = 0.74, *p* = 0.66, η^2^*_p_* = 0.01). Table 2 also displays the episodic memory results. The experimental group had higher episodic memory scores at both the baseline (19.22 vs. 17.20) and follow-up (18.15 vs. 15.77). There was a significant main effect for time (F = 13.70, *p* = 0.001, η^2^*_p_* = 0.27), but there was no significant group–time interaction (F = 0.27, *p* = 0.60, η^2^*_p_* = 0.01) or main effect for group (F = 1.00, *p* = 0.32, η^2^*_p_* = 0.03) (Figure 2).

## 4. Discussion

Memory function is critical for optimal daily functioning. The potential enhancement of memory function from exercise has numerous clinical implications for various populations including healthy adults and those with neurological conditions such as Alzheimer’s disease and Parkinson’s disease [50,51,52]. Among these populations, the enhancement of memory may help to facilitate future exercise behavior and help to attenuate the decline in memory function [53,54].

Extending this emerging exercise neurobiology field, the present experiment evaluated whether acute moderate-intensity exercise was associated with iconic and episodic memory function. Our results provided suggestive evidence that acute moderate-intensity exercise was associated with better iconic and episodic memory function including both short- and long-term memory. Notably, however, although the exercise group had better mean iconic and episodic memory scores when compared to the control group, these aggregate differences did not reach statistical significance. Our relatively small sample size may have contributed to statistical power issues. However, numerous other related experiments on exercise and memory have been adequately powered with the same sample size employed in our experiment [16,17,18,40]. Of course, relying strictly on statistical significance is a major research concern [55,56].

Given the novelty of this exercise and iconic memory paradigm, it would be of interest for future work to continue to evaluate this model. Of particular interest would be to evaluate whether an intensity-specific effect is observable [57], particularly for iconic memory. Perhaps moderate-intensity walking was not sufficient to induce an optimal level of arousal, and in turn, psychological attention. Of course, it is likely that too high of an intensity, with too short of a recovery period, may have the opposite effect by inducing psychological fatigue, and in turn, impairing iconic memory. Unlike the present study, other work on this topic may wish to standardize the intensity of the exercise bout to minimize between-subject differences in the exercise stimulus. Further work on this topic should also carefully consider the control task. Our control participants engaged in a Sudoku puzzle which, in theory, could have primed their performance on our evaluated memory tasks, and in turn, possibly masked any potential differences between the groups.

Although the present experiment is the first to our knowledge to evaluate the effects of acute exercise on iconic memory, this study extends other related work that has evaluated the effects of acute exercise on spatial-based memory, which includes a visual component like the present iconic memory task. In fact, meta-analytic research demonstrates that memory sub-type may moderate the effects of acute exercise on memory, and acute exercise is more strongly associated with short-term visuo-spatial memory when compared to verbal-auditory memory [19]. Our present episodic memory findings, although not statistically significant, converge with other work, suggesting that acute exercise may be associated with both short- and long-term episodic memory [15,18].

In conclusion, the present study evaluated the experimental effects of acute moderate-intensity aerobic exercise on iconic memory, short-term episodic, and long-term episodic memory. Our findings provide some suggestive evidence hinting towards an iconic memory and episodic benefit from acute exercise engagement. Future confirmatory work, particularly for iconic memory, is warranted. Future work on this topic should also aim to determine what magnitude of change in these memory parameters is clinically meaningful.

## Figures and Tables

**Table 1:** Characteristics of the study variables.

Variable	Exercise (*n* = 20)	Control (*n* = 20)
Age, mean years	21.0 (1.0)	20.8 (0.9)
% Female	75.0	55.0
% white	85.0	65.0
Waist circumference, mean cm	87.6 (13.6)	84.2 (9.6)
MVPA, mean min/week	198.0 (155.2)	224.1 (193.4)
Heart Rate, mean		
Resting	73.8 (12.5)	68.2 (12.3)
Midpoint	124.9 (22.7)	-
Endpoint	126.8 (23.6)	-
Post	84.2 (15.9)	68.9 (11.8)
Speed, mean mph	3.6 (0.1)	-

MVPA, Moderate to vigorous physical activity. Values in parentheses are SD estimates.

**Table 2:** Memory scores (iconic memory, mean) across the experimental and control groups.

Variable	Exercise (*n* = 20)		Control (*n* = 20)	
	Trial 1	Trial 2	Trial 1	Trial 2
Iconic memory, mean				
100 ms				
Number correct	1.95 (1.3)	2.70 (0.9)	2.10 (1.0)	2.55 (0.8)
Number of mislocation errors	0.45 (0.6)	0.45 (0.6)	0.25 (0.5)	0.60 (1.0)
Number of intrusion errors	0.65 (1.3)	0.35 (0.8)	0.20 (0.5)	0.10 (0.3)
200 ms				
Number correct	3.05 (1.0)	3.15 (1.0)	3.10 (0.7)	2.95 (0.8)
Number of mislocation errors	0.85 (0.9)	0.45 (0.7)	0.45 (0.6)	0.45 (0.7)
Number of intrusion errors	0.25 (0.7)	0.35 (0.7)	0.10 (0.3)	0.25 (0.6)
300 ms				
Number correct	3.80 (1.1)	3.55 (1.0)	3.25 (0.9)	3.40 (0.9)
Number of mislocation errors	0.40 (0.9)	0.70 (0.9)	0.50 (0.8)	0.60 (1.0)
Number of intrusion errors	0.35 (0.9)	0.40 (0.8)	0.30 (0.5)	0.25 (0.44)
500 ms				
Number correct	4.90 (1.4)	4.90 (1.2)	4.40 (1.2)	4.50 (1.4)
Number of mislocation errors	0.40 (0.8)	0.85 (1.1)	0.30 (0.7)	0.20 (0.5)
Number of intrusion errors	0.45 (0.9)	0.25 (0.5)	0.30 (0.7)	0.10 (0.3)
800 ms				
Number correct	4.10 (1.3)	5.80 (1.3)	4.35 (1.4)	5.85 (1.0)
Number of mislocation errors	1.05 (1.6)	0.55 (1.0)	0.65 (1.0)	0.10 (0.4)
Number of intrusion errors	0.30 (0.5)	0.50 (0.8)	0.40 (0.8)	0.30 (0.8)
Episodic memory, mean				
Short-term	19.22 (6.7)		17.20 (7.9)	
Long-term	18.15 (6.2)		15.77 (7.3)	

## Supplementary Materials

Available online at http://www.mdpi.com/2077-0383/7/6/146/s1. Supplementary Materials A. Schematic of the experimental protocol; Supplementary Materials B. Episodic Memory Passage; Supplementary Materials C. Scoring units for episodic memory passage.
