# Supplementary material for: Experimental Effects of Acute Exercise on Iconic Memory, Short-Term Episodic, and Long-Term Episodic Memory
Source: J Clin Med. 2018 Jun 11;7(6):146. doi: 10.3390/jcm7060146 (PMC6024998; doi:10.3390/jcm7060146)
Supplement: Supplementary file 1 [file jcm-07-00146-s001.pdf]

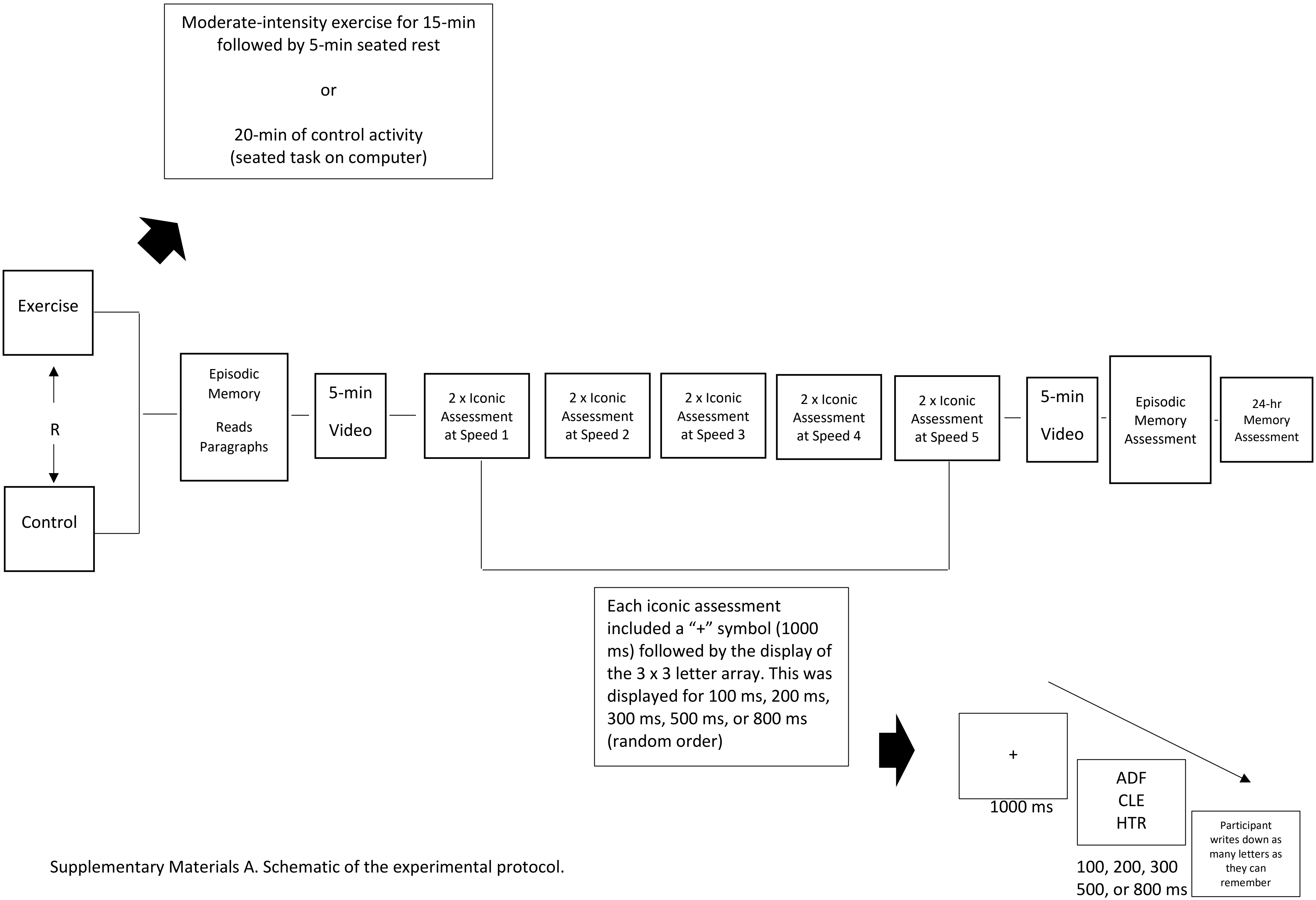

Supplementary Materials A. Schematic of the experimental protocol.

Supplementary Materials B. Episodic Memory Passage.

The two boys ran until they came to the driveway. “See, I told you today was good for skipping school,” said Mark. “Mom is never home on Thursday,” he added. Tall hedges hid the house from the road so the pair strolled across the finely landscaped yard. “I never knew your place was so big,” said Pete. “Yeah, but it’s nicer now than it used to be since Dad had the new stone siding put on and added the fireplace.”

There were front and back doors and a side door which led to the garage which was empty except for three parked 10-speed bikes. They went in the side door, Mark explaining that it was always open in case his younger sisters got home earlier than their mother.

Pete wanted to see the house so Mark started with the living room. It, like the rest of the downstairs, was newly painted. Mark turned on the stereo, the noise of which worried Pete. “Don’t worry, the nearest house is a quarter of a mile away,” Mark shouted. Pete felt more comfortable observing that no houses could be seen in any direction beyond the huge yard.

The dining room, with all the china, silver and cut glass, was no place to play so the boys moved into the kitchen where they made sandwiches. Mark said they would not go to the basement because it had been damp and musty ever since the new plumbing had been installed.

“This is where my Dad keeps his famous paintings and his coin collection,” Mark said as they peered into the den. Mark bragged that he could get spending money whenever he needed it since he discovered that his Dad kept a lot in the desk drawer.

There were three upstairs bedrooms. Mark showed Pete his mother’s closet which was filled with furs and the locked box which held her jewels. His sisters’ room was uninteresting except for the color TV which Mark carried to his room. Mark bragged that the bathroom in the hall was his since one had been added to his sisters’ room for their use. The big highlight in his room, though, was a leak in the ceiling where the old roof had finally rotted.

Supplementary Materials C. Scoring units for episodic memory passage.

Give them 1 point for each of the details they note below

Burglar Units

1. Tall hedges hide house.
2. Mom not home (on Thursday.)
3. Garage empty except for (3–10 speed) bikes.
4. Side door unlocked (in case sisters get home early).
5. There is a stereo
6. The nearest house is (1/4 mile) away Or there are no nearby houses.
7. There is china.
8. There is silver.
9. There is cut glass.
10. (The den has) paintings.
11. (The den has) a coin collection.
12. (Dad keeps) cash in his desk.
13. (Mom has a box with) Jewels
14. Mom has fine furs.
15. There is a (colour) T.V.

Homebuyer Units

1. The yard is finely landscaped.
2. The house is big.
3. There is new stone siding.
4. There is a new fireplace.
5. The downstairs area is newly painted.
6. The basement is damp.
7. The basement is musty/smells
8. The basement has new plumbing
9. There are three bedrooms
10. (The sisters' room has) a new bathroom.
11. There is a hall bathroom/There are two bathrooms.
12. There is a leak in Marks ceiling
13. The roof has rotted.

Neutral Units

1. The boys ran to the driveway.
2. The boys are skipping school.
3. One boy is named Mark.
4. One boy is named Pete.
5. Mark gives Pete a tour of the house.
6. Mark shows Pete the living room.
7. The boys listen to the stereo/Pete's worried about the stereo's noise.
8. Marks shows Pete the dining room.
9. The boys make sandwiches (in the Kitchen).
10. Mark shows Pete the kitchen.
11. Mark shows Pete the den.

12. Mark shows Pete the Parents’ room.
13. Mark’s sisters’ room is boring.
14. Mark took the TV from his sisters’ room.
